# Unrelenting Fear Under Stress: Neural Circuits and Mechanisms for the Immediate Extinction Deficit

**DOI:** 10.3389/fnsys.2022.888461

**Published:** 2022-04-19

**Authors:** Stephen Maren

**Affiliations:** Department of Psychological and Brain Sciences, Institute for Neuroscience, Texas A&M University, College Station, TX, United States

**Keywords:** extinction, stress, fear conditioning, norepinephrine, locus coeruleus, amygdala, infralimbic cortex, PTSD–posttraumatic stress disorder

## Abstract

Therapeutic interventions for disorders of fear and anxiety rely on behavioral approaches that reduce pathological fear memories. For example, learning that threat-predictive stimuli are no longer associated with aversive outcomes is central to the extinction of conditioned fear responses. Unfortunately, fear memories are durable, long-lasting, and resistant to extinction, particularly under high levels of stress. This is illustrated by the “immediate extinction deficit,” which is characterized by a poor long-term reduction of conditioned fear when extinction procedures are attempted within hours of fear conditioning. Here, I will review recent work that has provided new insight into the neural mechanisms underlying resistance to fear extinction. Emerging studies reveal that locus coeruleus norepinephrine modulates amygdala-prefrontal cortical circuits that are critical for extinction learning. These data suggest that stress-induced activation of brain neuromodulatory systems biases fear memory at the expense of extinction learning. Behavioral and pharmacological strategies to reduce stress in patients undergoing exposure therapy might improve therapeutic outcomes.

## Introduction

Survival depends on detecting and defending against threats in the environment. These functions, shaped over millennia by natural selection, rely on specialized neural and behavioral adaptations. For example, when a rat detects a predator, it will draw from a repertoire of species-specific defense responses that include freezing, jumping, fleeing, and biting (Bolles, 1970). The nature of this response depends on several factors, particularly the proximity of the predator and the imminence of the threat (Fanselow and Lester, 1988; Perusini and Fanselow, 2015). Once danger has passed animals disengage from defense behaviors to resume other activities. But yielding to safety in the immediate aftermath of threat is risky business. Animals are justifiably conservative when it comes to the prospect of losing their lives. A rat can afford to lose a meal or two, but it cannot afford to be caught by a predator. Maintaining a defensive posture after a dangerous encounter is highly adaptive.

Of course, animals must not only defend against present danger, but they must also learn to anticipate future threats should they survive. Decades of work have described brain circuits that rapidly encode and store memories of an aversive experience to ward off future threats (Davis, 1992; LeDoux, 2000; Maren, 2001; Maren and Quirk, 2004; Orsini and Maren, 2012; Maren et al., 2013; Herry and Johansen, 2014; Janak and Tye, 2015; Tovote et al., 2015). Learning is not only central to mobilizing defensive behavior to potential threats, but also in selecting the specific defensive responses an animal employs in a particular situation (Moscarello and Maren, 2018). In addition to learning about the circumstances that predict danger, animals also rapidly encode escape routes and shelters used to evade threats. Memories for the spatial location of safe havens allow for rapid escape responses when threat is encountered (Vale et al., 2017; Evans et al., 2018). Of course, learning that is reinforced by threat reduction should not interfere with consolidating memories for recently experienced threats. The brain systems and cellular mechanisms dedicated to storing fear memories should be resistant to disruption soon after a dangerous encounter. Surviving future threats requires that these memories be lifelong and indelible.

Several forms of learning are recruited to encode memories for aversive events. For example, intense noxious stimuli rapidly potentiate and sensitize behavioral responses they elicit (Borszcz et al., 1989; Boulis and Davis, 1989; Gewirtz et al., 1998). Sensitization is a non-associative form of learning that is short-lived (minutes to hours) and is not stimulus-specific: behavioral responses to many different innocuous stimuli are increased by sensitization (Groves and Thompson, 1970). Associative learning, on the other hand, results in relatively specific and durable long-term memories (hours to years) of aversive events and the circumstances (including cues and contexts) that predict them (Rescorla and Holland, 1982; Wasserman and Miller, 1997; Pearce and Bouton, 2001). An example of this form of learning is Pavlovian fear conditioning, in which animals acquire conditioned fear responses (CRs, such as freezing behavior) to innocuous conditioned stimuli (CSs, such as auditory tones) paired with aversive unconditioned stimuli (USs, such as a footshock). Pavlovian fear memories are rapidly acquired, exceptionally enduring, and highly resistant to disruption once learned. Not surprisingly, this type of learning has been the subject of intense investigation. It is not only essential to the adaptive function of defensive behavior systems, but also involved in the etiology of disorders of fear and anxiety in humans.

The durability of fear memory has been underscored by decades of work on another learning process: extinction. During experimental extinction procedures, CSs are repeatedly presented to the animal in the absence of the US (Pavlov, 1927; Konorski, 1967; Rescorla, 2000; Bouton et al., 2021). Consequently, animals learn that the CS no longer predicts the US, and this leads to a reduction in the frequency and vigor of conditioned responses. But the suppression of conditional responding after extinction is labile: CRs return under several conditions (Bouton, 1993; Bouton et al., 2006, 2021). For example, the mere passage of time leads to spontaneous recovery of extinguished CRs and presenting an extinguished CS outside the extinction context leads to a renewal of conditional responding. These relapse phenomena not only demonstrate that extinction memories are labile, but also that fear memories are very durable and difficult to disrupt (Goode and Maren, 2014, 2019; Goode et al., 2017).

Over 15 years ago, we discovered more evidence for the durability of fear memory. We found that extinction procedures performed soon after fear conditioning failed to yield long-term suppression of conditioned fear (Maren and Chang, 2006; Chang and Maren, 2009). We termed this phenomenon the “immediate extinction deficit” or IED. Considerable work has confirmed this finding and the IED has now been described in several species and paradigms (Schiller et al., 2008; Woods and Bouton, 2008; Huff et al., 2009; Kim et al., 2010; Golkar and Öhman, 2012; MacPherson et al., 2013; Stafford et al., 2013; Merz et al., 2016). Several lines of evidence suggest that the IED results from the stress associated with the conditioning procedure (Maren, 2014). Consistent with this possibility, deficits in “delayed” extinction learning have been reported after a variety of acute or chronic stressors (Izquierdo et al., 2006; Miracle et al., 2006; Chauveau et al., 2012; Knox et al., 2012; Maroun et al., 2013; Hartley et al., 2014; Maren and Holmes, 2016).

From an evolutionary perspective, resistance to extinction under stress serves to protect recently acquired fear memories from disruption and functions as a critical adaptation that permits animals to survive future threats. However, the resistance of fear memories to extinction poses challenges for therapeutic interventions for fear and anxiety disorders in humans. These interventions rely on extinction-based procedures such as prolonged exposure therapy and are susceptible to relapse (Norrholm et al., 2008; Kearns et al., 2012; Vervliet et al., 2013; Craske et al., 2018). For this reason, it is essential to understand the psychological and neurobiological mechanisms underlying stress-induced impairments in fear extinction. Here, I will review recent work on the neural and behavioral mechanisms of the immediate extinction deficit. This work reveals brain systems that operate to constrain extinction learning in the immediate aftermath of an aversive experience. Understanding the nature of these constraints on extinction learning has important implications for optimizing therapeutic interventions for humans, particularly soon after trauma.

## Recent Fear Is Resistant to Extinction

It has long been known that memories are labile soon after they are encoded (Lechner et al., 1999; McGaugh, 2000). A wide variety of neural insults cause severe amnesia for events occurring minutes to hours (and occasionally months to years) prior to the injury. This provides strong support for the view that newly acquired information requires the passage of time to become consolidated as a long-term memory. Decades of work have elucidated the neural mechanisms of underlying memory consolidation and have shown that a host of cellular processes, including protein synthesis, are required to stabilize long-term memories within hours of their encoding (Schafe et al., 2001; Alberini, 2005; Redondo and Morris, 2011). Beyond this initial phase of cellular consolidation, brain systems that represent memory (including fear memories) also reorganize over time (Frankland and Bontempi, 2005; Tonegawa et al., 2018; Josselyn and Tonegawa, 2020). Both cellular and systems consolidation processes represent periods during which memories are vulnerable to disruption after encoding.

**FIGURE 1:**
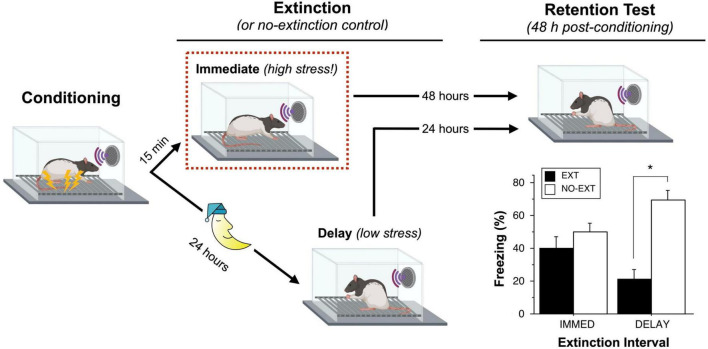
Recent fear is resistant to extinction. Maren and Chang (2006) compared groups of rats that underwent an extinction (EXT) procedure either 15 min or 24 h after auditory fear conditioning; each of these groups was compared to a group of animals that did not undergo extinction (NO-EXT). During the extinction session, rats in the IMMED condition exhibit high levels of sensitized fear (high stress) prior to the first extinction trial, whereas rats in the DELAY condition exhibit low levels of freezing (low stress). Despite showing similar levels of within-session extinction, rats in the IMMED condition exhibit poor long-term retention of extinction relative to animals in the DELAY condition. Unlike DELAY rats, rats in the IMMED condition exhibited similar and high levels of conditioned freezing compared to non-extinguished controls. Elements of the figure were created with BioRender.com; the data shown in the plot were previously published (Maren and Chang, 2006).

Because of these vulnerabilities, we imagined that behavioral manipulations, such as extinction, might be particularly effective if delivered shortly after conditioning. To test this hypothesis, we submitted male rats to an auditory fear conditioning procedure and followed that by an extinction procedure (45 CS-alone trials) either 15 min or 24 h after conditioning (Figure 1; Maren and Chang, 2006). Critically, we included control groups at each time point that did not undergo extinction training so that we could determine the relative effectiveness of each extinction procedure. Consistent with established work, we found that delivering extinction trials 24 h after conditioning (a delayed extinction procedure) was effective at suppressing conditioned freezing. However, delivering extinction trials 15 min after conditioning (an immediate extinction procedure) resulted in poor extinction retrieval. This immediate extinction deficit persisted for several hours after conditioning and was evident even after over 200 massed extinction trials. Although conditioned freezing declined during the extinction session, this reduction was found to be short-lived and likely due to habituation—conditioned freezing showed near complete recovery within 24 h of the immediate extinction procedure (Chang and Maren, 2009).

Additional experiments suggested that the footshock stress associated with the conditioning procedure was responsible for the IED. For example, we found that delivering unsignaled footshock immediately prior to a delayed (24 h) extinction session impaired later extinction retrieval (Maren and Chang, 2006). Similarly, limiting conditioned freezing before an immediate extinction session by reducing the number of conditioning trials or US intensity eliminates the IED (Maren and Chang, 2006; Jo and Choi, 2020). These data suggest that the non-associative sensitization of fear associated with footshock may be a factor that contributes to the IED. Consistent with context-independence of sensitization (Davis, 1989), we have observed that the IED occurs both in the conditioning context as well as in different contexts in which non-associative freezing is expressed soon after conditioning (Maren and Chang, 2006; Chang and Maren, 2009). Although the majority of work on the IED has been performed in male rats, recent work indicates that the IED is comparable in male and female rats (Binette et al., 2022).

Although several groups have observed the IED, there are some notable exceptions. Using auditory fear conditioning and extinction procedures similar to those of Maren and Chang (2006) and Ponnusamy et al. (2016) found that immediate extinction procedures produced significant decreases in freezing relative to non-extinguished controls. Interestingly, baseline freezing prior to the first extinction trial was relatively low (and no different than that in the delayed control group). This suggests that the high level of arousal that has been suggested to be requisite for the IED as not obtained in this study. Archbold et al. (2010) also failed to obtain an IED after contextual fear conditioning, although this was primarily due to particularly weak extinction retention in the delayed extinction condition. Lastly, Myers et al. (2006) also explored the effects of an immediate extinction procedure in a fear-potentiated startle paradigm in rats. Surprisingly, they found that delivering extinction trials 10 min after fear conditioning resulted in *weaker* relapse of conditioned fear. Relapse was indexed by the return of conditioned responding that is normally observed with the passage of time (spontaneous recovery), testing the CS outside the extinction context (renewal), or intervening exposure to the unconditioned stimulus (reinstatement). Based on these findings, the authors concluded that the immediate extinction procedure disrupted the consolidation of fear memory, produced long-term reductions in the expression of conditioned fear and eliminated fear relapse. However, close inspection of the data reveals that the lack of relapse observed after immediate extinction was not due to superior extinction, but just the opposite: conditioned fear was, in fact, resistant to immediate extinction. Because the animals did not acquire an extinction memory, they were unable to show relapse.

Interestingly, the IED has been observed after appetitive conditioning (Woods and Bouton, 2008) and in human fear conditioning experiments that use relatively weak unconditioned stimuli (Schiller et al., 2008; Huff et al., 2009; Merz et al., 2016) or non-emotional tasks (Merz and Wolf, 2019). These data have challenged the view that sensitization of fear in aversive conditioning experiments is necessary for the IED. However, it is possible that when extinction closely follows conditioning in the same physical context, the extinction trials are encoded within the interoceptive context of conditioning (produced by food or shock), a context that is absent at the time of extinction retrieval testing. This shift in interoceptive context might therefore lead to renewal of conditioned responding in animals that underwent immediate extinction or delayed extinction after a US reminder. However, matching the state of arousal driven by conditioning at the time of retrieval testing does not prevent expression of the IED (Woods and Bouton, 2008), although an experience with shock before extinction training reduces fear suppression as previously observed (Maren and Chang, 2006). These data suggest that the relative recency of conditioning to post-extinction retrieval testing (Devenport, 1998) does not account for the IED. An alternative idea is that the temporal gap between fear conditioning and extinction serves as an event boundary that segments and reduces interference between the two contingencies (Devenport, 1998; Dunsmoor et al., 2018). To test this hypothesis, we examined whether delivering extinction trials within seconds of conditioning (using the same ISI at which conditioning trials were delivered) would reduce the IED (Totty et al., 2019). However, we found that the continuous extinction procedure yielded a robust IED suggesting that a temporal gap between conditioning and extinction is not responsible for the IED.

Although the precise behavioral mechanisms underlying the IED are still uncertain, the existing data are most compatible with the possibility that conditioning results in emotional arousal or stress (whether aversive or appetitive) that impedes extinction learning. This conclusion is perhaps most strongly supported by recent data showing that pharmacological treatments that limit stress-sensitive neuromodulatory circuits in the brain attenuate the IED.

## Stress Modulates Neural Circuits Critical for Extinction Learning

Extinction procedures engage multiple neural circuits that mediate not only the retrieval and expression of conditioned responses, but also the encoding of prediction errors and contextual stimuli associated with the extinction contingency (Bouton et al., 2006, 2021; Radulovic and Tronson, 2010; Maren et al., 2013). Brainstem circuits are critical for encoding prediction errors that drive extinction learning and they convey this information to forebrain circuits that encode context-dependent extinction memories (McNally et al., 2011; Iordanova et al., 2021). A critical hub for extinction learning is centered in the medial prefrontal cortex (mPFC) and its connections with the hippocampus (HPC) and amygdala (Maren and Quirk, 2004; Quirk and Mueller, 2008; Orsini and Maren, 2012; Maren et al., 2013; Giustino and Maren, 2015; Bouton et al., 2021). Specifically, projections from the infralimbic (IL) division of the mPFC to the basolateral amygdala (BLA) and amygdala intercalated cells are implicated in the acquisition and expression of extinction (Likhtik et al., 2005, 2008; Bukalo et al., 2015; Do-Monte et al., 2015; Bloodgood et al., 2018; Hagihara et al., 2021). IL projections to the HPC via the thalamic nucleus reuniens are also critical for extinction learning and retrieval (Ramanathan et al., 2018; Ramanathan and Maren, 2019). In turn, reciprocal projections within these networks are involved in extinction retrieval and the suppressing of conditional responding (Senn et al., 2014; Jin and Maren, 2015; Marek et al., 2018; Silva et al., 2021).

Considerable work indicates that both acute and chronic stressors regulate extinction learning by modulating neural activity within hippocampal-prefrontal-amygdala networks (Maren and Holmes, 2016; Singewald and Holmes, 2019). Aversive stressors activate central and peripheral hormonal and neuromodulatory systems that drive sympathetic arousal and modulate activity within this stress-sensitive network. This response is coordinated by the hypothalamic-pituitary-adrenal axis, which regulates the release of adrenal glucocorticoids, and the brainstem locus coeruleus (LC), which is the primary source for forebrain norepinephrine (NE) (Jones and Moore, 1977; Lipski and Grace, 2013; Aston-Jones and Waterhouse, 2016; Poe et al., 2020; Ross and Van Bockstaele, 2020). Not surprisingly, both of these neuromodulatory systems have been implicated in aversive conditioning and extinction (Pugh et al., 1997; Korte, 2001; Rodrigues et al., 2009; Giustino et al., 2016b; Giustino and Maren, 2018; de Quervain et al., 2019; Likhtik and Johansen, 2019). For example, it is well-established that aversive footshock increases both Fos expression and spike firing in LC neurons (Rasmussen et al., 1986; Abercrombie and Jacobs, 1987; Grant et al., 1992; Chiang and Aston-Jones, 1993; Rassnick et al., 1998), as well as increasing NE release in forebrain efferents, including the amygdala (Galvez et al., 1996; Quirarte et al., 1998) and mPFC (Gresch et al., 1994; Goldstein et al., 1996; Jedema et al., 1999). Indeed, the role for stress-induced activation of forebrain noradrenergic activity in promoting the acquisition and consolidation of aversive learning and memory has long been appreciated (Ferry et al., 1999; Johansen et al., 2014; McGaugh, 2015; Díaz-Mataix et al., 2017; Schiff et al., 2017; Giustino and Maren, 2018). However, other forms of learning are opposed by stress, including Pavlovian extinction (Raio and Phelps, 2015; Maren and Holmes, 2016; Singewald and Holmes, 2019).

This paradox might be explained by the non-linear (inverted-U) function relating noradrenergic activity to learning and memory (Gold et al., 1975; Snyder et al., 2012). Either abnormally low or excessive activation of the LC-NE system can impair learning and memory processes, including extinction (Arnsten, 2009; Fitzgerald et al., 2015; Giustino and Maren, 2018). From an evolutionary perspective, the LC-NE system has been adapted to both promote the rapid encoding of aversive memories under stress and enable the extinction of those memories when danger has passed. By this view, excessive activation of the LC-NE system under high levels of stress promotes fear memory encoding at the expense of extinction, whereas modest levels of LC-NE system engagement under relatively low-stress conditions promotes extinction learning (Giustino et al., 2016b; Giustino and Maren, 2018).

## Noradrenergic Antagonists Rescue the Immediate Extinction Deficit

Footshock stress results in sustained LC activation and NE release in forebrain regions critical for fear conditioning and extinction. Importantly, shock-induced activation of the LC-NE system may undermine extinction learning in the immediate aftermath of fear conditioning, resulting in the IED. Consistent with this idea, there is extensive evidence that β-adrenergic receptor antagonists, such as propranolol, reduce stress-induced fear responses in both rodents and humans (Cole and Koob, 1988; Fitzgerald et al., 2014; Chalkia et al., 2019). Systemic administration of propranolol reduces the expression of conditioned freezing (Rodriguez-Romaguera et al., 2009; Fitzgerald et al., 2015; Giustino et al., 2020; Leal Santos et al., 2021) and reduces increases in conditioned fear induced by corticotropin-releasing factor (CRF) (Cole and Koob, 1988). If a high fear state associated with recent footshock opposes extinction, then pharmacologically limiting this state with systemic propranolol should attenuate the IED and facilitate extinction under stress.

To explore this possibility, we administered propranolol immediately after auditory fear conditioning in male rats undergoing an immediate extinction procedure in which CS-alone trials were presented 15 min after the last conditioning trial (Fitzgerald et al., 2015). Consistent with previous reports, we found that systemic propranolol reduced post-shock freezing in the interval prior to extinction training. In addition to a reduction in post-shock freezing, propranolol facilitated the reduction in freezing behavior that occurred during the immediate extinction procedure. Importantly, this facilitation of extinction was long-lasting. Although control rats undergoing immediate extinction exhibited a near-complete recovery of conditioned freezing 24 h later, rats receiving propranolol exhibited a robust attenuation of conditioned freezing the following day. The facilitation of extinction under stress was not due to a propranolol-induced impairment in the consolidation of the conditioned fear memory. Rats that received immediate post-shock propranolol in the absence of extinction training showed high levels of CS-elicited freezing that were not different from vehicle-treated controls (Fitzgerald et al., 2015). This reveals that propranolol resulted in long-term decreases in freezing by augmenting extinction learning, rather than impairing the consolidation of fear conditioning. It is important to note that although propranolol administration facilitated immediate extinction, we also found that it impaired delayed extinction (Mueller et al., 2008; Uematsu et al., 2017). This suggests that noradrenergic transmission may be involved in promoting extinction learning during basal, “low stress” conditions (delayed extinction), while it undermines extinction under high levels of stress (immediate extinction). Of course, this begs the question: how does propranolol rescue the immediate extinction deficit?

Decades of work have revealed that the IL cortex, a mPFC region that is interconnected with both the HPC and amygdala, is a critical neural substrate underlying the extinction of conditioned fear (Maren and Quirk, 2004; Delgado et al., 2006; Quirk et al., 2006; Quirk and Mueller, 2008; Giustino and Maren, 2015). Neuronal activity within the IL correlates with suppression of conditioned freezing (Milad and Quirk, 2002; Chang et al., 2010; Wilber et al., 2011; Giustino et al., 2016a,^2019^), electrical or optogenetic activation of the IL can facilitate extinction learning (Milad et al., 2004; Do-Monte et al., 2015) and pharmacological or optogenetic inhibition of neuronal activity in the IL impairs extinction learning (Laurent and Westbrook, 2009; Sierra-Mercado et al., 2011; Do-Monte et al., 2015). In addition, chronic stress remodels (Izquierdo et al., 2006; Miracle et al., 2006) and reduces the excitability (Wilber et al., 2011) of IL principal neurons causing impairments in forming long-term extinction memories, particularly when the stress occurs prior to fear conditioning (Chakraborty and Chattarji, 2019). Importantly, both synaptic and dendritic changes can occur within hours of an acute stressor (Chen et al., 2008; Yuen et al., 2009; Nava et al., 2017), which suggests that suppression of IL activity by footshock may cause the IED. To test this hypothesis, we explored spontaneous single-unit firing in the mPFC after auditory fear conditioning in rats. We found that conditioning induced a dramatic, but short-lived, increase in spontaneous spike firing in both the prelimbic (PL) and IL cortices (Fitzgerald et al., 2015). Interestingly, this conditioning-induced potentiation of spike firing was followed by an extended suppression of neuronal activity in the IL (Fitzgerald et al., 2015; Giustino et al., 2016a). This suppression of spontaneous firing in the IL was coincident with the emergence of post-shock freezing behavior and was maximal roughly 30 min after the final footshock (a period during which the IED is readily obtained); it was also most robust following signaled shocks (Fitzgerald et al., 2015; Giustino et al., 2016a). Suppression of spontaneous spike firing persisted for up to 60 min (the duration of the recording session) in many IL units.

Because systemic propranolol administration rescues the IED, we hypothesized that it might promote extinction under stress by regulating post-shock spike firing in the mPFC. Consistent with this possibility, rats that received systemic propranolol injections prior to auditory fear conditioning exhibited much smaller perturbations of mPFC spike firing than vehicle-treated rats (Fitzgerald et al., 2015). Specifically, propranolol blunted both the immediate post-shock increases in PL and IL spike firing and eliminated the sustained suppression of spike firing observed in the IL during the 60-min post-shock period. These changes in spike firing were paralleled by reductions in post-shock freezing in propranolol-treated rats. Importantly, however, changes in mPFC spike firing were dissociable from freezing behavior. Auditory CSs produced dramatic increases in conditioned freezing behavior 24 h after conditioning, but this was not associated with the same increases in mPFC spike firing that were previously observed immediately after footshock (Fitzgerald et al., 2015). Together, these results suggest that stress-induced activation of the LC-NE system suppresses the activity of a key cortical substrate involved in extinction learning. The post-shock suppression of spontaneous spike firing in the IL appears to underlie, at least in part, the IED.

## Neural Circuit for Noradrenergic Regulation of Extinction Learning Under Stress

Systemic propranolol administration may regulate neuronal activity by direct effects on β-adrenergic receptors in IL or by affecting a brain region that regulates IL activity. For example, it is well-known that NE promotes aversive memory formation through its actions in the BLA, which, in turn, projects to and regulates IL function (Floresco and Tse, 2007; Dilgen et al., 2013; McGarry and Carter, 2016). Indeed, BLA projections to IL have been implicated in extinction learning (Senn et al., 2014). To explore whether β-adrenergic receptors in IL or BLA (or both) are involved in the IED, we made intracranial infusions of propranolol into these structures immediately after fear conditioning, a timepoint that was roughly 20 min prior to an immediate extinction procedure (Giustino et al., 2017). Unlike systemic propranolol infusions, neither IL nor BLA infusions reliably decreased post-shock freezing or freezing behavior during the immediate extinction procedure (Giustino et al., 2017). Interestingly, recent work suggests that noradrenergic regulation of post-shock freezing behavior may depend on LC projections to the central nucleus of the amygdala (CEA) (Gu et al., 2020). Importantly, intra-BLA propranolol infusions, but not infusions into the IL, promoted long-term extinction of conditioned freezing and reduced the IED. Rats treated with intra-BLA propranolol exhibited significantly lower levels of CS-evoked freezing during the extinction retrieval test 24 h after the immediate extinction procedure. This effect was not due to an effect of BLA propranolol on the consolidation of conditioned fear memory, because immediate post-shock intra-BLA propranolol infusions had no effect on the long-term retrieval of conditioned fear in the absence of extinction (Giustino et al., 2017). Hence, local antagonism of β-adrenergic receptors in the BLA, but not the IL, rescues the IED.

The BLA receives dense noradrenergic innervation from the LC (Jones and Moore, 1977; Asan, 1998; Zhang et al., 2013). This suggests that stress-induced activation of LC-NE projections to the BLA may be critical for regulating the IED. To test this hypothesis, we explored whether footshock stress alters neuronal activity in the BLA and whether these effects are modulated by the LC and depend on noradrenergic receptors in the BLA. In contrast to our previous observations in the mPFC, fear conditioning produced dramatic and sustained increases in the spontaneous activity of BLA neuronal activity (Giustino et al., 2020). Importantly, this shock-induced increase in BLA spike firing was completely attenuated by systemic propranolol administration. To determine whether BLA spike firing is modulated by LC activity, we expressed an excitatory “designer receptor exclusively activated by designer drug” (DREADD; AAV-PRSx8-hM3Dq) in LC noradrenergic neurons. We then chemogenetically activated these neurons with the synthetic DREADD ligand clozapine-*N*-oxide (CNO) and coupled activation of LC with the delivery of weak footshock. Weak footshocks alone produced minimal increases in BLA spike firing that were dramatically potentiated when delivered concomitant with chemogenetic activation of LC-NE neurons (Giustino et al., 2020). This suggests that shock-elicited NE release drive increases in BLA firing in behaving rats. Although the LC-NE system has been reported to have primarily inhibitory effects on BLA spike firing (Chen and Sara, 2007), LC stimulation and iontophoretic application of NE can increase spiking in a subset of BLA neurons (Buffalari and Grace, 2007). Moreover, fear conditioning increases BLA excitability by reducing NE-driven GABAergic inhibition of BLA neurons without affecting noradrenergic facilitation of BLA excitability (Skelly et al., 2017, 2016). These results suggest that sustained footshock-induced activation of LC leads to a persistent increase in BLA excitability that underlies the IED.

Having identified a role for LC-NE activation in shock-induced increases in BLA neuronal firing, we next explored the contribution of the LC to the IED. We reasoned that an IED would not normally be induced by conditioning with weak footshocks, but that this deficit would become apparent after pairing a weak conditioning protocol with chemogenetic LC activation. Chemogenetic LC activation robustly increased conditioned freezing during the immediate extinction procedure as previously observed (Giustino et al., 2020). Consistent with our hypothesis, we also found that chemogenetic activation of the LC promoted an IED under conditions that do not typically lead to a stress-induced extinction deficit. Specifically, rats treated with vehicle after the immediate extinction procedure showed reliable reductions in conditioned freezing during a drug-free extinction retrieval test (relative to non-extinguished controls). This revealed that the weak conditioning procedure failed to produce an IED. In contrast, CNO-treated rats undergoing the immediate extinction procedure exhibited poor extinction retrieval (high freezing) and were no different than non-extinguished controls during the drug-free retrieval test (Giustino et al., 2020). Hence, chemogenetic activation of the LC-NE system drove an extinction learning deficit under weak conditioning procedures that are not normally sufficient to yield the IED.

**FIGURE 2:**
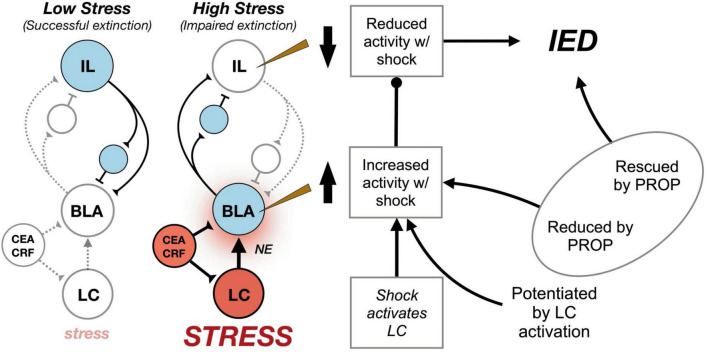
Circuit model for the immediate extinction deficit (IED). Under basal conditions (“Low Stress”) delayed extinction procedures conducted 24 h after fear conditioning recruit infralimbic (IL) cortical circuits (blue) that mediate the acquisition and expression of extinction learning. Inhibition of conditioned fear is presumed to arise from IL-mediated excitation of inhibitory interneurons (small circles) that reduce the excitability of basolateral amygdala (BLA) principal cells representing fear memories. However, delivering extinction trials soon after fear conditioning, when animals are under extreme stress (“High Stress”) results in activation of locus coeruleus (LC) noradrenergic neurons (red) that release norepinephrine (NE) in forebrain targets, including the BLA (blue). Neurons in the central amygdala (CEA) release corticotropin-releasing hormone (CRF) in the LC and BLA to facilitate stress-induced activation of these brain areas. Consequently, fear conditioning dramatically increases spontaneous spike firing in the BLA, while decreasing spike firing in IL. We speculate that BLA decreases IL spike firing by activating IL interneurons (small circles) and driving feed-forward inhibition in IL principal cells. Shock-induced increases in BLA firing are modulated by the LC, and LC activation during weak shock enables the IED when it would not normally occur. Systemic or intra-BLA administration of the β-adrenergic antagonist, propranolol (PROP), attenuates the IED and limits shock-elicited changes in BLA and IL spike firing (Fitzgerald et al., 2015; Giustino et al., 2016a,^2017^, ^2020^).

Finally, we sought to determine whether the induction of an IED by LC-NE activation was mediated by β-adrenergic receptors on the BLA. To this end, we infused vehicle or propranolol into the BLA prior to systemic CNO (or vehicle) injections in rats expressing excitatory DREADDs in the LC (Giustino et al., 2020). Animals then underwent a weak conditioning protocol followed by an immediate extinction procedure. The animals that received CNO infusions exhibited a robust IED as previously observed. Importantly, this CNO-induced IED was blocked by intra-BLA propranolol infusions. Propranolol infusions alone did not affect conditional freezing. Collectively, these data suggest that the IED is mediated by stress-induced activation of LC-NE afferents in the BLA, which in turn potentiates BLA firing and impedes the extinction of fear. Moreover, these results suggest that BLA projections to the IL may play a critical role in the shock-induced changes in IL spike firing that are associated with the IED. Indeed, IL principal neurons experience strong feed-forward inhibition by cortical interneurons after the activation of excitatory BLA afferents (Floresco and Tse, 2007; Dilgen et al., 2013; McGarry and Carter, 2016). These results suggest a model whereby acute stressors activate LC-NE projections to BLA, thereby elevating BLA activity to promote the stabilization of fear memory. We propose that stress-induced increases in BLA firing yield a sustained inhibition of neuronal activity in the IL and impair extinction of fear memories (Figure 2).

## Corticotropin-Releasing Factor System Modulates Neural Circuits Underlying the Immediate Extinction Deficit

In addition to the strong efferent projections to the BLA, the LC-NE system also receives a potent descending projection from the CEA (Van Bockstaele et al., 1998; Ross and Van Bockstaele, 2020). Importantly, footshock increases neuronal activity in the CEA (Radulovic et al., 1998; Yu et al., 2017; Giovanniello et al., 2020) and optogenetic activation of CEA induces high tonic firing rates in LC neurons (McCall et al., 2015). Considerable work indicates that CEA-mediated increases in LC spike firing are mediated by the neuropeptide hormone, CRF (McCall et al., 2015). Hence, reciprocal projections between the LC and amygdala function to promote feed-forward excitation within these brain areas. Specifically, LC activation drives NE-dependent increases in BLA and CEA excitability and CEA-CRF neurons (which are also excited by BLA afferents) increase the excitability of LC projection neurons (Van Bockstaele et al., 1998; Valentino and Van Bockstaele, 2008).

The excitatory effect of the CEA on tonic LC-NE firing suggests that CEA-CRF neurons may be involved in the IED. To explore this issue, Jo et al. (2020) used transgenic mouse models to either silence CEA-CRF neurons or inactivate CRF production from these cells. In both cases, silencing CRF neurons or inactivating CRF attenuated the IED: mice exhibited normal conditioning and within-session extinction of conditioned freezing but failed to exhibit long-term suppression of freezing behavior. Temporary chemogenetic inhibition of CEA-CRF neurons also facilitated immediate extinction, whereas CNO-mediated excitation of these neurons produced deficits in a normally effective delayed extinction procedure. Interestingly, the activity of CEA-CRF neurons differed under immediate and delayed extinction procedures. Recordings of calcium signals in CEA-CRF neurons (expressing GCaMP6m) revealed elevated and sustained CS-induced responses in mice undergoing immediate extinction. Interestingly, elevated CS-evoked responses in animals undergoing immediate extinction persisted during the extinction retrieval test (Jo et al., 2020). Although the spontaneous activity of CEA-CRF neurons was not quantified in non-extinguished animals, it is intriguing to speculate that footshock stress might tonically elevate the activity of these cells. Nonetheless, these data suggest that increased CS-elicited activity in CEA-CRF neurons may drive the IED. Consistent with this, Jo et al. (2020) found that optogenetic inhibition of CEA-CRF neurons during the CS also attenuated the IED. Although not directly tested, these data suggest that CEA-CRF projections to the LC may be involved in the IED.

The stress-induced activation of CEA has also been suggested to increase CRF levels in the BLA (Roozendaal et al., 2002). Moreover, the BLA has a high density of CRF receptors (De Souza et al., 1985) and CRF increases the excitability of BLA neurons (Rainnie et al., 1992). Hence, it is possible that CRF drives increases in BLA excitability after footshock stress (Giustino et al., 2020) and contributes to the IED. To test this hypothesis, Hollis et al. (2016) infused a CRFR1 antagonist into the BLA prior to an immediate extinction procedure in rats. The drug dose-dependently attenuated the IED indexed by conditioned freezing during a drug-free extinction retrieval test 24 h after extinction (Hollis et al., 2016). This effect was not due to a suppression of freezing *per se* insofar as the drug did not affect freezing during the immediate extinction session. Moreover, a no-extinction control group revealed that the drug did not exert its effect by disrupting the consolidation of fear memory. In contrast, local infusion of a CRF agonist into the BLA prior to a delayed extinction procedure results in an extinction impairment (Abiri et al., 2014; Hollis et al., 2016). Interestingly, CRF was shown to exert its actions in the BLA through the modulation of the phosphorylation state of AMPA-type glutamate receptors (Hollis et al., 2016). This suggests that an attenuation of local synaptic plasticity in the BLA contributes to the IED. Together, these results suggest that CRF has both local and circuit effects that contribute to shock-induced increases in BLA excitability that drive the IED. Indeed, CRF may act synergistically to antagonize extinction learning soon after fear conditioning by both direct actions on BLA CRF receptors and indirect activation of LC-NE projections to the BLA. An interesting area for future work is to determine how sex differences in CRH regulation of LC function (Bangasser et al., 2010; Bangasser and Cuarenta, 2021) affect stress-induced impairments in extinction learning.

## Conclusion

Fear conditioning is an adaptive form of learning that allows an animal to mobilize defensive behaviors in anticipation of threat. Aversive memories are rapidly encoded, long-lasting, and susceptible to relapse after conditioning. Critically, fear memories are highly resistant to extinction soon after they are encoded, resulting in an IED when extinction trials are delivered within hours of fear conditioning. A growing body of evidence reveals that the IED is mediated by the recruitment of a stress-sensitive network that prioritizes the encoding and consolidation of fear memory even when the prevailing threat has passed. Stress-induced impairments in extinction learning involve activation of the LC-NE system, which appears to regulate amygdalo-prefrontal circuits involved in extinction learning. Specifically, we propose that noradrenergic activation of the BLA recruits mPFC-projecting neurons that functionally inhibit IL principal cells involved in extinction learning. We imagine that this is supported by BLA afferents on mPFC interneurons, which provide potent feed-forward inhibition of mPFC principal cells. In addition to the LC-NE system, CRF-containing neurons in the CEA play a critical role in the IED. Descending CEA→LC projections may amplify shock-induced increases in LC excitability, potentiate NE effects on BLA principal cells, and drive feed-forward excitation of BLA neuronal activity that protects fear memory from extinction under stress.

The role for the LC-NE system in mediating resistance to extinction under stress has important implications for understanding disorders of fear and anxiety (Fitzgerald et al., 2014; Giustino et al., 2016b). Patients with post-traumatic stress disorder (PTSD), for example, exhibit dysregulated noradrenergic neurotransmission (Grillon et al., 1996; Southwick et al., 1999; Geracioti et al., 2001; Pitman et al., 2012) and resistance to extinction (Wessa and Flor, 2007; Milad et al., 2009; VanElzakker et al., 2014). Importantly, noradrenergic hyperarousal may impede therapeutic interventions, such as prolonged exposure therapy, that rely upon extinction learning to reduce pathological fear (McCurry et al., 2020). There is evidence that treatment with propranolol after reactivating trauma memory can reduce symptom severity in patients with PTSD (Vaiva et al., 2003; Hoge et al., 2012; Brunet et al., 2018) or specific phobias (Soeter and Kindt, 2015). Potential mechanisms for these effects include the suppression of fear expression, facilitation of extinction, or impaired reconsolidation of fear memories (Grillon et al., 2004; Kindt et al., 2009; Kroes et al., 2010, 2016; Maren, 2011; Soeter and Kindt, 2015; Chalkia et al., 2019). Preclinical work on the IED suggests that propranolol might be most effective in patients with noradrenergic hyperarousal, particularly when combined with cognitive-behavioral therapy. Ultimately, deciphering the neural circuits and cellular mechanisms underlying stress-impaired extinction offers considerable promise for understanding both adaptive regulation of emotional behavior and pathological disorders of fear and anxiety.

## Author Contributions

SM wrote the manuscript.

## Conflict of Interest

The author declares that the research was conducted in the absence of any commercial or financial relationships that could be construed as a potential conflict of interest.

## Publisher’s Note

All claims expressed in this article are solely those of the authors and do not necessarily represent those of their affiliated organizations, or those of the publisher, the editors and the reviewers. Any product that may be evaluated in this article, or claim that may be made by its manufacturer, is not guaranteed or endorsed by the publisher.

## References

[B1] Abercrombie E. D. Jacobs B. L. (1987). Single-unit response of noradrenergic neurons in the locus coeruleus of freely moving cats. I. Acutely presented stressful and nonstressful stimuli. *J. Neurosci.* 7 2837–2843. 10.1523/JNEUROSCI.07-09-02837.1987 3625275 PMC6569145

[B2] Abiri D. Douglas C. E. Calakos K. C. Barbayannis G. Roberts A. Bauer E. P. (2014). Fear extinction learning can be impaired or enhanced by modulation of the CRF system in the basolateral nucleus of the amygdala. *Behav. Brain Res.* 271 234–239. 10.1016/j.bbr.2014.06.021 24946071 PMC5126972

[B3] Alberini C. M. (2005). Mechanisms of memory stabilization: are consolidation and reconsolidation similar or distinct processes? *Trends Neurosci.* 28 51–56. 10.1016/j.tins.2004.11.001 15626497

[B4] Archbold G. E. B. Bouton M. E. Nader K. (2010). Evidence for the persistence of contextual fear memories following immediate extinction. *Eur. J. Neurosci.* 31 1303–1311. 10.1111/j.1460-9568.2010.07161.x 20345921

[B5] Arnsten A. F. T. (2009). Stress signalling pathways that impair prefrontal cortex structure and function. *Nat. Rev. Neurosci.* 10 410–422. 10.1038/nrn2648 19455173 PMC2907136

[B6] Asan E. (1998). The catecholaminergic innervation of the rat amygdala. *Adv. Anat. Embryol. Cell Biol.* 142 1–118. 10.1007/978-3-642-72085-7 9586282

[B7] Aston-Jones G. Waterhouse B. (2016). Locus coeruleus: From global projection system to adaptive regulation of behavior. *Brain Res.* 1645 75–78. 10.1016/j.brainres.2016.03.001 26969408 PMC4969192

[B8] Bangasser D. A. Cuarenta A. (2021). Sex differences in anxiety and depression: circuits and mechanisms. *Nat. Rev. Neurosci.* 22 674–684. 10.1038/s41583-021-00513-0 34545241

[B9] Bangasser D. A. Curtis A. Reyes B. A. S. Bethea T. T. Parastatidis I. Ischiropoulos H. (2010). Sex differences in corticotropin-releasing factor receptor signaling and trafficking: potential role in female vulnerability to stress-related psychopathology. *Mol. Psychiatr.* 15 896–904. 10.1038/mp.2010.66 20548297 PMC2935505

[B10] Binette A. N. Totty M. S. Maren S. (2022). Sex differences in the immediate extinction deficit and renewal of extinguished fear in rats. *BioRxiv* [preprint]. 10.1101/2022.02.17.480946 PMC9187087 35687598

[B11] Bloodgood D. W. Sugam J. A. Holmes A. Kash T. L. (2018). Fear extinction requires infralimbic cortex projections to the basolateral amygdala. *Transl. Psychiatry* 8:60. 10.1038/s41398-018-0106-x 29507292 PMC5838104

[B12] Bolles R. C. (1970). Species-specific defense reactions and avoidance learning. *Psychol. Rev.* 77 32–48. 10.1037/h0028589

[B13] Borszcz G. Cranney J. Leaton R. (1989). Influence of long-term sensitization on long-term habituation of the acoustic startle response in rats: central gray lesions, preexposure, and extinction. *J. Exp. Psychol.* 15 54–64. 10.1037/0097-7403.15.1.54 2926335

[B14] Boulis N. M. Davis M. (1989). Footshock-induced sensitization of electrically elicited startle reflexes. *Behav. Neurosci.* 103 504–508. 10.1037/0735-7044.103.3.504 2544200

[B15] Bouton M. E. (1993). Context, time, and memory retrieval in the interference paradigms of Pavlovian learning. *Psychol. Bull.* 114 80–99. 10.1037/0033-2909.114.1.80 8346330

[B16] Bouton M. E. Maren S. McNally G. P. (2021). Behavioral and neurobiological mechanisms of pavlovian and instrumental extinction learning. *Physiol. Rev.* 101 611–681. 10.1152/physrev.00016.2020 32970967 PMC8428921

[B17] Bouton M. E. Westbrook R. F. Corcoran K. A. Maren S. (2006). Contextual and temporal modulation of extinction: behavioral and biological mechanisms. *Biol. Psychiatry* 60 352–360. 10.1016/j.biopsych.2005.12.015 16616731

[B18] Brunet A. Saumier D. Liu A. Streiner D. L. Tremblay J. Pitman R. K. (2018). Reduction of PTSD Symptoms With Pre-Reactivation Propranolol Therapy: A Randomized Controlled Trial. *Am. J. Psychiatry* 175 427–433. 10.1176/appi.ajp.2017.17050481 29325446

[B19] Buffalari D. M. Grace A. A. (2007). Noradrenergic modulation of basolateral amygdala neuronal activity: opposing influences of alpha-2 and beta receptor activation. *J. Neurosci.* 27 12358–12366. 10.1523/JNEUROSCI.2007-07.2007 17989300 PMC6673273

[B20] Bukalo O. Pinard C. R. Silverstein S. Brehm C. Hartley N. D. Whittle N. (2015). Prefrontal inputs to the amygdala instruct fear extinction memory formation. *Sci. Adv.* 1:e1500251. 10.1126/sciadv.1500251 26504902 PMC4618669

[B21] Chakraborty P. Chattarji S. (2019). Timing is everything: differential effects of chronic stress on fear extinction. *Psychopharmacology* 236 73–86. 10.1007/s00213-018-5053-y 30306227

[B22] Chalkia A. Weermeijer J. Van Oudenhove L. Beckers T. (2019). Acute but not permanent effects of propranolol on fear memory expression in humans. *Front. Hum. Neurosci.* 13:51. 10.3389/fnhum.2019.00051 30846933 PMC6394213

[B23] Chang C. Berke J. D. Maren S. (2010). Single-unit activity in the medial prefrontal cortex during immediate and delayed extinction of fear in rats. *PLoS One* 5:e11971. 10.1371/journal.pone.0011971 20700483 PMC2916837

[B24] Chang C. Maren S. (2009). Early extinction after fear conditioning yields a context-independent and short-term suppression of conditional freezing in rats. *Learn. Mem.* 16 62–68. 10.1101/lm.1085009 19141467 PMC2632848

[B25] Chauveau F. Lange M. D. Jüngling K. Lesting J. Seidenbecher T. Pape H.-C. (2012). Prevention of stress-impaired fear extinction through neuropeptide s action in the lateral amygdala. *Neuropsychopharmacology* 37 1588–1599. 10.1038/npp.2012.3 22298122 PMC3358750

[B26] Chen F. J. Sara S. J. (2007). Locus coeruleus activation by foot shock or electrical stimulation inhibits amygdala neurons. *Neuroscience* 144 472–481. 10.1016/j.neuroscience.2006.09.037 17097235

[B27] Chen Y. Dubé C. M. Rice C. J. Baram T. Z. (2008). Rapid loss of dendritic spines after stress involves derangement of spine dynamics by corticotropin-releasing hormone. *J. Neurosci.* 28 2903–2911. 10.1523/JNEUROSCI.0225-08.2008 18337421 PMC2409370

[B28] Chiang C. Aston-Jones G. (1993). Response of locus coeruleus neurons to footshock stimulation is mediated by neurons in the rostral ventral medulla. *Neuroscience* 53 705–715. 10.1016/0306-4522(93)90618-p 8487951

[B29] Cole B. J. Koob G. F. (1988). Propranolol antagonizes the enhanced conditioned fear produced by corticotropin releasing factor. *J. Pharmacol. Exp. Ther.* 247 902–910. 2849675

[B30] Craske M. G. Hermans D. Vervliet B. (2018). State-of-the-art and future directions for extinction as a translational model for fear and anxiety. *Philos. Trans. R. Soc. Lond. B, Biol. Sci.* 373:20170025. 10.1098/rstb.2017.0025 29352025 PMC5790824

[B31] Davis M. (1989). Sensitization of the acoustic startle reflex by footshock. *Behav. Neurosci.* 103 495–503. 10.1037/0735-7044.103.3.495 2736065

[B32] Davis M. (1992). The role of the amygdala in fear and anxiety. *Annu. Rev. Neurosci.* 15 353–375. 10.1146/annurev.ne.15.030192.002033 1575447

[B33] de Quervain D. Wolf O. T. Roozendaal B. (2019). Glucocorticoid-induced enhancement of extinction-from animal models to clinical trials. *Psychopharmacology* 236 183–199. 10.1007/s00213-018-5116-0 30610352 PMC6373196

[B34] De Souza E. B. Insel T. R. Perrin M. H. Rivier J. Vale W. W. Kuhar M. J. (1985). Corticotropin-releasing factor receptors are widely distributed within the rat central nervous system: an autoradiographic study. *J. Neurosci.* 5 3189–3203. 10.1523/JNEUROSCI.05-12-03189.1985 3001239 PMC6565229

[B35] Delgado M. R. Olsson A. Phelps E. A. (2006). Extending animal models of fear conditioning to humans. *Biol. Psychol.* 73 39–48. 10.1016/j.biopsycho.2006.01.006 16472906

[B36] Devenport L. D. (1998). Spontaneous recovery without interference: Why remembering is adaptive. *Anim Learn Behav.* 26 172–181. 10.3758/BF03199210

[B37] Díaz-Mataix L. Piper W. T. Schiff H. C. Roberts C. H. Campese V. D. Sears R. M. (2017). Characterization of the amplificatory effect of norepinephrine in the acquisition of Pavlovian threat associations. *Learn. Mem.* 24 432–439. 10.1101/lm.044412.116 28814469 PMC5580522

[B38] Dilgen J. Tejeda H. A. O’Donnell P. (2013). Amygdala inputs drive feedforward inhibition in the medial prefrontal cortex. *J. Neurophysiol.* 110 221–229. 10.1152/jn.00531.2012 23657281 PMC3727030

[B39] Do-Monte F. H. Manzano-Nieves G. Quiñones-Laracuente K. Ramos-Medina L. Quirk G. J. (2015). Revisiting the role of infralimbic cortex in fear extinction with optogenetics. *J. Neurosci.* 35 3607–3615. 10.1523/JNEUROSCI.3137-14.2015 25716859 PMC4339362

[B40] Dunsmoor J. E. Kroes M. C. W. Moscatelli C. M. Evans M. D. Davachi L. Phelps E. A. (2018). Event segmentation protects emotional memories from competing experiences encoded close in time. *Nat. Hum. Behav.* 2 291–299. 10.1038/s41562-018-0317-4 30221203 PMC6136428

[B41] Evans D. A. Stempel A. V. Vale R. Ruehle S. Lefler Y. Branco T. (2018). A synaptic threshold mechanism for computing escape decisions. *Nature* 558 590–594. 10.1038/s41586-018-0244-6 29925954 PMC6235113

[B42] Fanselow M. S. Lester L. S. (1988). “A functional behavioristic approach to aversively motivated behavior: Predatory imminence as a determinant of the topography of defensive behavior,” in *Evolution and learning*, eds Bolles R. C. Beecher M. D. (Mahwah: Lawrence Erlbaum Associates, Inc), 185–212.

[B43] Ferry B. Roozendaal B. McGaugh J. L. (1999). Role of norepinephrine in mediating stress hormone regulation of long-term memory storage: a critical involvement of the amygdala. *Biol. Psychiatry* 46 1140–1152. 10.1016/s0006-3223(99)00157-2 10560021

[B44] Fitzgerald P. J. Giustino T. F. Seemann J. R. Maren S. (2015). Noradrenergic blockade stabilizes prefrontal activity and enables fear extinction under stress. *Proc. Natl. Acad. Sci. USA* 112 E3729–E3737. 10.1073/pnas.1500682112 26124100 PMC4507202

[B45] Fitzgerald P. J. Seemann J. R. Maren S. (2014). Can fear extinction be enhanced? A review of pharmacological and behavioral findings. *Brain Res. Bull.* 105 46–60. 10.1016/j.brainresbull.2013.12.007 24374101 PMC4039692

[B46] Floresco S. B. Tse M. T. (2007). Dopaminergic regulation of inhibitory and excitatory transmission in the basolateral amygdala-prefrontal cortical pathway. *J. Neurosci.* 27 2045–2057. 10.1523/JNEUROSCI.5474-06.2007 17314300 PMC6673549

[B47] Frankland P. W. Bontempi B. (2005). The organization of recent and remote memories. *Nat. Rev. Neurosci.* 6 119–130. 10.1038/nrn1607 15685217

[B48] Galvez R. Mesches M. H. McGaugh J. L. (1996). Norepinephrine release in the amygdala in response to footshock stimulation. *Neurobiol. Learn. Mem.* 66 253–257. 10.1006/nlme.1996.0067 8946419

[B49] Geracioti T. D. Baker D. G. Ekhator N. N. West S. A. Hill K. K. Bruce A. B. (2001). CSF norepinephrine concentrations in posttraumatic stress disorder. *Am. J. Psychiatry* 158 1227–1230. 10.1176/appi.ajp.158.8.1227 11481155

[B50] Gewirtz J. McNish K. Davis M. (1998). Lesions of the bed nucleus of the stria terminalis block sensitization of the acoustic startle reflex produced by repeated stress, but not fear-potentiated startle. *Prog. Neuropsychopharmacol. Biol. Psychiatr.* 22 625–648. 10.1016/s0278-5846(98)00028-1 9682277

[B51] Giovanniello J. Yu K. Furlan A. Nachtrab G. T. Sharma R. Chen X. (2020). A Central Amygdala-Globus Pallidus Circuit Conveys Unconditioned Stimulus-Related Information and Controls Fear Learning. *J. Neurosci.* 40 9043–9054. 10.1523/JNEUROSCI.2090-20.2020 33067362 PMC7673004

[B52] Giustino T. F. Fitzgerald P. J. Maren S. (2016b). Revisiting propranolol and PTSD: Memory erasure or extinction enhancement? *Neurobiol. Learn. Mem.* 130 26–33. 10.1016/j.nlm.2016.01.009 26808441 PMC4818733

[B53] Giustino T. F. Fitzgerald P. J. Maren S. (2016a). Fear expression suppresses medial prefrontal cortical firing in rats. *PLoS One* 11:e0165256. 10.1371/journal.pone.0165256 27776157 PMC5077087

[B54] Giustino T. F. Fitzgerald P. J. Ressler R. L. Maren S. (2019). Locus coeruleus toggles reciprocal prefrontal firing to reinstate fear. *Proc. Natl. Acad. Sci. USA* 116 8570–8575. 10.1073/pnas.1814278116 30971490 PMC6486780

[B55] Giustino T. F. Maren S. (2015). The role of the medial prefrontal cortex in the conditioning and extinction of fear. *Front. Behav. Neurosci.* 9:298. 10.3389/fnbeh.2015.00298 26617500 PMC4637424

[B56] Giustino T. F. Maren S. (2018). Noradrenergic modulation of fear conditioning and extinction. *Front. Behav. Neurosci.* 12:43. 10.3389/fnbeh.2018.00043 29593511 PMC5859179

[B57] Giustino T. F. Ramanathan K. R. Totty M. S. Miles O. W. Maren S. (2020). Locus Coeruleus Norepinephrine Drives Stress-Induced Increases in Basolateral Amygdala Firing and Impairs Extinction Learning. *J. Neurosci.* 40 907–916. 10.1523/JNEUROSCI.1092-19.2019 31801809 PMC6975297

[B58] Giustino T. F. Seemann J. R. Acca G. M. Goode T. D. Fitzgerald P. J. Maren S. (2017). β-Adrenoceptor Blockade in the Basolateral Amygdala, But Not the Medial Prefrontal Cortex, Rescues the Immediate Extinction Deficit. *Neuropsychopharmacology* 42 2537–2544. 10.1038/npp.2017.89 28462941 PMC5686500

[B59] Gold P. E. van Buskirk R. B. McGaugh J. L. (1975). Effects of hormones on time-dependent memory storage processes. *Prog. Brain Res.* 42 210–211. 10.1016/s0079-6123(08)63665-1 172964

[B60] Goldstein L. E. Rasmusson A. M. Bunney B. S. Roth R. H. (1996). Role of the amygdala in the coordination of behavioral, neuroendocrine, and prefrontal cortical monoamine responses to psychological stress in the rat. *J. Neurosci.* 16 4787–4798. 10.1523/JNEUROSCI.16-15-04787.1996 8764665 PMC6579011

[B61] Golkar A. Öhman A. (2012). Fear extinction in humans: effects of acquisition-extinction delay and masked stimulus presentations. *Biol. Psychol.* 91 292–301. 10.1016/j.biopsycho.2012.07.007 22898744

[B62] Goode T. D. Holloway-Erickson C. M. Maren S. (2017). Extinction after fear memory reactivation fails to eliminate renewal in rats. *Neurobiol. Learn. Mem.* 142 41–47. 10.1016/j.nlm.2017.03.001 28274824 PMC5457330

[B63] Goode T. D. Maren S. (2014). Animal models of fear relapse. *ILAR J.* 55 246–258. 10.1093/ilar/ilu008 25225304 PMC4197897

[B64] Goode T. D. Maren S. (2019). Common neurocircuitry mediating drug and fear relapse in preclinical models. *Psychopharmacology* 236 415–437. 10.1007/s00213-018-5024-3 30255379 PMC6373193

[B65] Grant S. J. Bittman K. Benno R. H. (1992). Both phasic sensory stimulation and tonic pharmacological activation increase Fos-like immunoreactivity in the rat locus coeruleus. *Synapse* 12 112–118. 10.1002/syn.890120204 1481134

[B66] Gresch P. J. Sved A. F. Zigmond M. J. Finlay J. M. (1994). Stress-induced sensitization of dopamine and norepinephrine efflux in medial prefrontal cortex of the rat. *J. Neurochem.* 63 575–583. 10.1046/j.1471-4159.1994.63020575.x 8035182

[B67] Grillon C. Cordova J. Morgan C. A. Charney D. S. Davis M. (2004). Effects of the beta-blocker propranolol on cued and contextual fear conditioning in humans. *Psychopharmacology* 175 342–352. 10.1007/s00213-004-1819-5 15007536

[B68] Grillon C. Southwick S. M. Charney D. S. (1996). The psychobiological basis of posttraumatic stress disorder. *Mol. Psychiatry* 1 278–297. 9118351

[B69] Groves P. M. Thompson R. F. (1970). Habituation: a dual-process theory. *Psychol. Rev.* 77 419–450. 10.1037/h0029810 4319167

[B70] Gu Y. Piper W. T. Branigan L. A. Vazey E. M. Aston-Jones G. Lin L. (2020). A brainstem-central amygdala circuit underlies defensive responses to learned threats. *Mol. Psychiatry* 25 640–654. 10.1038/s41380-019-0599-6 31758092 PMC7042728

[B71] Hagihara K. M. Bukalo O. Zeller M. Aksoy-Aksel A. Karalis N. Limoges A. (2021). Intercalated amygdala clusters orchestrate a switch in fear state. *Nature* 594 403–407. 10.1038/s41586-021-03593-1 34040259 PMC8402941

[B72] Hartley C. A. Gorun A. Reddan M. C. Ramirez F. Phelps E. A. (2014). Stressor controllability modulates fear extinction in humans. *Neurobiol. Learn. Mem.* 113 149–156. 10.1016/j.nlm.2013.12.003 24333646 PMC4053478

[B73] Herry C. Johansen J. P. (2014). Encoding of fear learning and memory in distributed neuronal circuits. *Nat. Neurosci.* 17 1644–1654. 10.1038/nn.3869 25413091

[B74] Hoge E. A. Worthington J. J. Nagurney J. T. Chang Y. Kay E. B. Feterowski C. M. (2012). Effect of acute posttrauma propranolol on PTSD outcome and physiological responses during script-driven imagery. *CNS Neurosci Ther.* 18 21–27. 10.1111/j.1755-5949.2010.00227.x 22070357 PMC6493400

[B75] Hollis F. Sevelinges Y. Grosse J. Zanoletti O. Sandi C. (2016). Involvement of CRFR1 in the basolateral amygdala in the immediate fear extinction deficit. *eNeuro* 3:ENEURO.84–ENEURO.16. 10.1523/ENEURO.0084-16.2016 27844053 PMC5093152

[B76] Huff N. C. Hernandez J. A. Blanding N. Q. LaBar K. S. (2009). Delayed extinction attenuates conditioned fear renewal and spontaneous recovery in humans. *Behav. Neurosci.* 123 834–843. 10.1037/a0016511 19634943 PMC2749460

[B77] Iordanova M. D. Yau J. O.-Y. McDannald M. A. Corbit L. H. (2021). Neural substrates of appetitive and aversive prediction error. *Neurosci. Biobehav. Rev.* 123 337–351. 10.1016/j.neubiorev.2020.10.029 33453307 PMC7933120

[B78] Izquierdo A. Wellman C. L. Holmes A. (2006). Brief uncontrollable stress causes dendritic retraction in infralimbic cortex and resistance to fear extinction in mice. *J. Neurosci.* 26 5733–5738. 10.1523/JNEUROSCI.0474-06.2006 16723530 PMC6675270

[B79] Janak P. H. Tye K. M. (2015). From circuits to behaviour in the amygdala. *Nature* 517 284–292. 10.1038/nature14188 25592533 PMC4565157

[B80] Jedema H. P. Sved A. F. Zigmond M. J. Finlay J. M. (1999). Sensitization of norepinephrine release in medial prefrontal cortex: effect of different chronic stress protocols. *Brain Res.* 830 211–217. 10.1016/s0006-8993(99)01369-4 10366677

[B81] Jin J. Maren S. (2015). Prefrontal-Hippocampal Interactions in Memory and Emotion. *Front. Syst. Neurosci.* 9:170. 10.3389/fnsys.2015.00170 26696844 PMC4678200

[B82] Jo K. I. Choi J. (2020). Immediate Extinction Renewal Deficit following Pavlovian Fear Conditioning with Mild, but not StrongFootshock Unconditioned Stimulus. *Kor. J. Cogn. Biol. Psychol.* 32 213–221.

[B83] Jo Y. S. Namboodiri V. M. K. Stuber G. D. Zweifel L. S. (2020). Persistent activation of central amygdala CRF neurons helps drive the immediate fear extinction deficit. *Nat. Commun.* 11:422. 10.1038/s41467-020-14393-y 31969571 PMC6976644

[B84] Johansen J. P. Diaz-Mataix L. Hamanaka H. Ozawa T. Ycu E. Koivumaa J. (2014). Hebbian and neuromodulatory mechanisms interact to trigger associative memory formation. *Proc. Natl. Acad. Sci. USA* 111 E5584–E5592. 10.1073/pnas.1421304111 25489081 PMC4280619

[B85] Jones B. E. Moore R. Y. (1977). Ascending projections of the locus coeruleus in the rat. *II. Autoradiograph. Stud. Brain Res.* 127 25–53. 10.1016/0006-8993(77)90378-X 301051

[B86] Josselyn S. A. Tonegawa S. (2020). Memory engrams: Recalling the past and imagining the future. *Science* 367:eaaw4325. 10.1126/science.aaw4325 31896692 PMC7577560

[B87] Kearns M. C. Ressler K. J. Zatzick D. Rothbaum B. O. (2012). Early interventions for PTSD: a review. *Depress. Anx.* 29 833–842. 10.1002/da.21997 22941845 PMC3665083

[B88] Kim S. C. Jo Y. S. Kim I. H. Kim H. Choi J.-S. (2010). Lack of medial prefrontal cortex activation underlies the immediate extinction deficit. *J. Neurosci.* 30 832–837. 10.1523/JNEUROSCI.4145-09.2010 20089891 PMC6633106

[B89] Kindt M. Soeter M. Vervliet B. (2009). Beyond extinction: erasing human fear responses and preventing the return of fear. *Nat. Neurosci.* 12 256–258. 10.1038/nn.2271 19219038

[B90] Knox D. George S. A. Fitzpatrick C. J. Rabinak C. A. Maren S. Liberzon I. (2012). Single prolonged stress disrupts retention of extinguished fear in rats. *Learn. Mem.* 19 43–49. 10.1101/lm.024356.111 22240323 PMC3262971

[B91] Konorski J. (1967). *Integrative Activity of the Brain: An Interdisciplinary Approach.* Chicago: University of Chicago Press.

[B92] Korte S. M. (2001). Corticosteroids in relation to fear, anxiety and psychopathology. *Neurosci. Biobehav. Rev.* 25 117–142. 10.1016/s0149-7634(01)00002-1 11323078

[B93] Kroes M. C. W. Strange B. A. Dolan R. J. (2010). Beta-adrenergic blockade during memory retrieval in humans evokes a sustained reduction of declarative emotional memory enhancement. *J. Neurosci.* 30 3959–3963. 10.1523/JNEUROSCI.5469-09.2010 20237266 PMC2935678

[B94] Kroes M. C. W. Tona K.-D. den Ouden H. E. M. Vogel S. van Wingen G. A. Fernández G. (2016). How Administration of the Beta-Blocker Propranolol Before Extinction can Prevent the Return of Fear. *Neuropsychopharmacology* 41 1569–1578. 10.1038/npp.2015.315 26462618 PMC4820039

[B95] Laurent V. Westbrook R. F. (2009). Inactivation of the infralimbic but not the prelimbic cortex impairs consolidation and retrieval of fear extinction. *Learn. Mem.* 16 520–529. 10.1101/lm.1474609 19706835

[B96] Leal Santos S. Stackmann M. Muñoz Zamora A. Mastrodonato A. De Landri A. V. Vaughan N. (2021). Propranolol decreases fear expression by modulating fear memory traces. *Biol. Psychiatry* 89 1150–1161. 10.1016/j.biopsych.2021.01.005 33766406 PMC8201901

[B97] Lechner H. A. Squire L. R. Byrne J. H. (1999). 100 years of consolidation–remembering Müller and Pilzecker. *Learn. Mem.* 6 77–87. 10.1101/lm.6.2.77 10327233

[B98] LeDoux J. E. (2000). Emotion circuits in the brain. *Annu. Rev. Neurosci.* 23 155–184. 10.1146/annurev.neuro.23.1.155 10845062

[B99] Likhtik E. Johansen J. P. (2019). Neuromodulation in circuits of aversive emotional learning. *Nat. Neurosci.* 22 1586–1597. 10.1038/s41593-019-0503-3 31551602

[B100] Likhtik E. Pelletier J. G. Paz R. Paré D. (2005). Prefrontal control of the amygdala. *J. Neurosci.* 25 7429–7437. 10.1523/JNEUROSCI.2314-05.2005 16093394 PMC6725290

[B101] Likhtik E. Popa D. Apergis-Schoute J. Fidacaro G. A. Paré D. (2008). Amygdala intercalated neurons are required for expression of fear extinction. *Nature* 454 642–645. 10.1038/nature07167 18615014 PMC2528060

[B102] Lipski W. J. Grace A. A. (2013). Footshock-induced responses in ventral subiculum neurons are mediated by locus coeruleus noradrenergic afferents. *Eur. Neuropsychopharmacol.* 23 1320–1328. 10.1016/j.euroneuro.2012.10.007 23394871 PMC3718869

[B103] MacPherson K. Whittle N. Camp M. Gunduz-Cinar O. Singewald N. Holmes A. (2013). Temporal factors in the extinction of fear in inbred mouse strains differing in extinction efficacy. *Biol. Mood Anxiety Disord.* 3 13. 10.1186/2045-5380-3-13 23830244 PMC3726460

[B104] Marek R. Jin J. Goode T. D. Giustino T. F. Wang Q. Acca G. M. (2018). Hippocampus-driven feed-forward inhibition of the prefrontal cortex mediates relapse of extinguished fear. *Nat. Neurosci.* 21 384–392. 10.1038/s41593-018-0073-9 29403033 PMC5957529

[B105] Maren S. (2001). Neurobiology of Pavlovian fear conditioning. *Annu. Rev. Neurosci.* 24 897–931. 10.1146/annurev.neuro.24.1.897 11520922

[B106] Maren S. (2011). Seeking a spotless mind: extinction, deconsolidation, and erasure of fear memory. *Neuron* 70 830–845. 10.1016/j.neuron.2011.04.023 21658578 PMC3112357

[B107] Maren S. (2014). Nature and causes of the immediate extinction deficit: a brief review. *Neurobiol. Learn. Mem.* 113 19–24. 10.1016/j.nlm.2013.10.012 24176924 PMC4004759

[B108] Maren S. Chang C. (2006). Recent fear is resistant to extinction. *Proc. Natl. Acad. Sci. USA* 103 18020–18025. 10.1073/pnas.0608398103 17090669 PMC1693865

[B109] Maren S. Holmes A. (2016). Stress and fear extinction. *Neuropsychopharmacology* 41 58–79. 10.1038/npp.2015.180 26105142 PMC4677122

[B110] Maren S. Phan K. L. Liberzon I. (2013). The contextual brain: implications for fear conditioning, extinction and psychopathology. *Nat. Rev. Neurosci.* 14 417–428. 10.1038/nrn3492 23635870 PMC5072129

[B111] Maren S. Quirk G. J. (2004). Neuronal signalling of fear memory. *Nat. Rev. Neurosci.* 5 844–852. 10.1038/nrn1535 15496862

[B112] Maroun M. Ioannides P. J. Bergman K. L. Kavushansky A. Holmes A. Wellman C. L. (2013). Fear extinction deficits following acute stress associate with increased spine density and dendritic retraction in basolateral amygdala neurons. *Eur. J. Neurosci.* 38 2611–2620. 10.1111/ejn.12259 23714419 PMC3773716

[B113] McCall J. G. Al-Hasani R. Siuda E. R. Hong D. Y. Norris A. J. Ford C. P. (2015). CRH Engagement of the Locus Coeruleus Noradrenergic System Mediates Stress-Induced Anxiety. *Neuron* 87 605–620. 10.1016/j.neuron.2015.07.002 26212712 PMC4529361

[B114] McCurry K. L. Frueh B. C. Chiu P. H. King-Casas B. (2020). Opponent Effects of Hyperarousal and Re-experiencing on Affective Habituation in Posttraumatic Stress Disorder. *Biol. Psychiatry Cogn. Neurosci. Neuroimaging* 5 203–212. 10.1016/j.bpsc.2019.09.006 31759868 PMC7010563

[B115] McGarry L. M. Carter A. G. (2016). Inhibitory gating of basolateral amygdala inputs to the prefrontal cortex. *J. Neurosci.* 36 9391–9406. 10.1523/JNEUROSCI.0874-16.2016 27605614 PMC5013187

[B116] McGaugh J. L. (2000). Memory–a century of consolidation. *Science* 287 248–251. 10.1126/science.287.5451.248 10634773

[B117] McGaugh J. L. (2015). Consolidating memories. *Annu. Rev. Psychol.* 66 1–24. 10.1146/annurev-psych-010814-014954 25559113

[B118] McNally G. P. Johansen J. P. Blair H. T. (2011). Placing prediction into the fear circuit. *Trends Neurosci.* 34 283–292. 10.1016/j.tins.2011.03.005 21549434 PMC4245078

[B119] Merz C. J. Hamacher-Dang T. C. Wolf O. T. (2016). Immediate extinction promotes the return of fear. *Neurobiol. Learn. Mem.* 131 109–116. 10.1016/j.nlm.2016.03.013 26995309

[B120] Merz C. J. Wolf O. T. (2019). The immediate extinction deficit occurs in a nonemotional learning paradigm. *Learn. Mem.* 26 39–45. 10.1101/lm.048223.118 30651376 PMC6340120

[B121] Milad M. R. Pitman R. K. Ellis C. B. Gold A. L. Shin L. M. Lasko N. B. (2009). Neurobiological basis of failure to recall extinction memory in posttraumatic stress disorder. *Biol. Psychiatry* 66 1075–1082. 10.1016/j.biopsych.2009.06.026 19748076 PMC2787650

[B122] Milad M. R. Quirk G. J. (2002). Neurons in medial prefrontal cortex signal memory for fear extinction. *Nature* 420 70–74. 10.1038/nature01138 12422216

[B123] Milad M. R. Vidal-Gonzalez I. Quirk G. J. (2004). Electrical stimulation of medial prefrontal cortex reduces conditioned fear in a temporally specific manner. *Behav. Neurosci.* 118 389–394. 10.1037/0735-7044.118.2.389 15113265

[B124] Miracle A. D. Brace M. F. Huyck K. D. Singler S. A. Wellman C. L. (2006). Chronic stress impairs recall of extinction of conditioned fear. *Neurobiol. Learn. Mem.* 85 213–218. 10.1016/j.nlm.2005.10.005 16337411

[B125] Moscarello J. M. Maren S. (2018). Flexibility in the face of fear: Hippocampal-prefrontal regulation of fear and avoidance. *Curr. Opin. Behav. Sci.* 19 44–49. 10.1016/j.cobeha.2017.09.010 29333482 PMC5764170

[B126] Mueller D. Porter J. T. Quirk G. J. (2008). Noradrenergic signaling in infralimbic cortex increases cell excitability and strengthens memory for fear extinction. *J. Neurosci.* 28 369–375. 10.1523/JNEUROSCI.3248-07.2008 18184779 PMC6670514

[B127] Myers K. M. Ressler K. J. Davis M. (2006). Different mechanisms of fear extinction dependent on length of time since fear acquisition. *Learn. Mem.* 13 216–223. 10.1101/lm.119806 16585797 PMC1409828

[B128] Nava N. Treccani G. Alabsi A. Kaastrup Mueller H. Elfving B. Popoli M. (2017). Temporal Dynamics of Acute Stress-Induced Dendritic Remodeling in Medial Prefrontal Cortex and the Protective Effect of Desipramine. *Cereb. Cortex* 27 694–705. 10.1093/cercor/bhv254 26523035

[B129] Norrholm S. D. Vervliet B. Jovanovic T. Boshoven W. Myers K. M. Davis M. (2008). Timing of extinction relative to acquisition: a parametric analysis of fear extinction in humans. *Behav. Neurosci.* 122 1016–1030. 10.1037/a0012604 18823159 PMC3731450

[B130] Orsini C. A. Maren S. (2012). Neural and cellular mechanisms of fear and extinction memory formation. *Neurosci. Biobehav. Rev.* 36 1773–1802. 10.1016/j.neubiorev.2011.12.014 22230704 PMC3345303

[B131] Pavlov I. P. (1927). *Conditioned Reflexes; an Investigation of the Physiological activity of the Cerebral Cortex.* London: Oxford University Press. 10.5214/ans.0972-7531.1017309 PMC4116985 25205891

[B132] Pearce J. M. Bouton M. E. (2001). Theories of associative learning in animals. *Annu. Rev. Psychol* 52 111–139. 10.1146/annurev.psych.52.1.111 11148301

[B133] Perusini J. N. Fanselow M. S. (2015). Neurobehavioral perspectives on the distinction between fear and anxiety. *Learn. Mem.* 22 417–425. 10.1101/lm.039180.115 26286652 PMC4561408

[B134] Pitman R. K. Rasmusson A. M. Koenen K. C. Shin L. M. Orr S. P. Gilbertson M. W. (2012). Biological studies of post-traumatic stress disorder. *Nat. Rev. Neurosci* 13 769–787. 10.1038/nrn3339 23047775 PMC4951157

[B135] Poe G. R. Foote S. Eschenko O. Johansen J. P. Bouret S. Aston-Jones G. (2020). Locus coeruleus: a new look at the blue spot. *Nat. Rev. Neurosci.* 21 644–659. 10.1038/s41583-020-0360-9 32943779 PMC8991985

[B136] Ponnusamy R. Zhuravka I. Poulos A. M. Shobe J. Merjanian M. Huang J. (2016). Retrieval and reconsolidation accounts of fear extinction. *Front. Behav. Neurosci.* 10:89. 10.3389/fnbeh.2016.00089 27242459 PMC4860411

[B137] Pugh C. R. Fleshner M. Rudy J. W. (1997). Type II glucocorticoid receptor antagonists impair contextual but not auditory-cue fear conditioning in juvenile rats. *Neurobiol. Learn. Mem.* 67 75–79. 10.1006/nlme.1996.3741 9013504

[B138] Quirarte G. L. Galvez R. Roozendaal B. McGaugh J. L. (1998). Norepinephrine release in the amygdala in response to footshock and opioid peptidergic drugs. *Brain Res.* 808 134–140. 10.1016/s0006-8993(98)00795-1 9767150

[B139] Quirk G. J. Garcia R. González-Lima F. (2006). Prefrontal mechanisms in extinction of conditioned fear. *Biol. Psychiatry* 60 337–343. 10.1016/j.biopsych.2006.03.010 16712801

[B140] Quirk G. J. Mueller D. (2008). Neural mechanisms of extinction learning and retrieval. *Neuropsychopharmacology* 33 56–72. 10.1038/sj.npp.1301555 17882236 PMC2668714

[B141] Radulovic J. Kammermeier J. Spiess J. (1998). Relationship between fos production and classical fear conditioning: effects of novelty, latent inhibition, and unconditioned stimulus preexposure. *J. Neurosci.* 18 7452–7461. 10.1523/JNEUROSCI.18-18-07452.1998 9736664 PMC6793227

[B142] Radulovic J. Tronson N. C. (2010). Molecular specificity of multiple hippocampal processes governing fear extinction. *Rev Neurosci* 21 1–17. 10.1515/revneuro.2010.21.1.1 20458884 PMC2922903

[B143] Rainnie D. G. Fernhout B. J. Shinnick-Gallagher P. (1992). Differential actions of corticotropin releasing factor on basolateral and central amygdaloid neurones, in vitro. *J. Pharmacol. Exp. Ther.* 263 846–858. 1331417

[B144] Raio C. M. Phelps E. A. (2015). The influence of acute stress on the regulation of conditioned fear. *Neurobiol. Stress* 1 134–146. 10.1016/j.ynstr.2014.11.004 25530986 PMC4268774

[B145] Ramanathan K. R. Jin J. Giustino T. F. Payne M. R. Maren S. (2018). Prefrontal projections to the thalamic nucleus reuniens mediate fear extinction. *Nat. Commun.* 9:4527. 10.1038/s41467-018-06970-z 30375397 PMC6207683

[B146] Ramanathan K. R. Maren S. (2019). Nucleus reuniens mediates the extinction of contextual fear conditioning. *Behav. Brain Res.* 374 112114. 10.1016/j.bbr.2019.112114 31351844 PMC6833945

[B147] Rasmussen K. Morilak D. A. Jacobs B. L. (1986). Single unit activity of locus coeruleus neurons in the freely moving cat. I. During naturalistic behaviors and in response to simple and complex stimuli. *Brain Res.* 371 324–334. 10.1016/0006-8993(86)90370-7 3697761

[B148] Rassnick S. Hoffman G. E. Rabin B. S. Sved A. F. (1998). Injection of corticotropin-releasing hormone into the locus coeruleus or foot shock increases neuronal Fos expression. *Neuroscience* 85 259–268. 10.1016/S0306-4522(97)00574-5 9607717

[B149] Redondo R. L. Morris R. G. M. (2011). Making memories last: the synaptic tagging and capture hypothesis. *Nat. Rev. Neurosci.* 12 17–30. 10.1038/nrn2963 21170072

[B150] Rescorla R. (2000). Experimental extinction. *Int. J. Psychol.* 35 109–109.

[B151] Rescorla R. A. Holland P. C. (1982). Behavioral Studies of Associative Learning in Animals. *Annu. Rev. Psychol.* 33 265–308. 10.1146/annurev.ps.33.020182.001405

[B152] Rodrigues S. M. LeDoux J. E. Sapolsky R. M. (2009). The influence of stress hormones on fear circuitry. *Annu. Rev. Neurosci.* 32 289–313. 10.1146/annurev.neuro.051508.135620 19400714

[B153] Rodriguez-Romaguera J. Sotres-Bayon F. Mueller D. Quirk G. J. (2009). Systemic propranolol acts centrally to reduce conditioned fear in rats without impairing extinction. *Biol. Psychiatr.* 65 887–892. 10.1016/j.biopsych.2009.01.009 19246030 PMC2695810

[B154] Roozendaal B. Brunson K. L. Holloway B. L. McGaugh J. L. Baram T. Z. (2002). Involvement of stress-released corticotropin-releasing hormone in the basolateral amygdala in regulating memory consolidation. *Proc. Natl. Acad. Sci. U.S.A* 99 13908–13913. 10.1073/pnas.212504599 12361983 PMC129796

[B155] Ross J. A. Van Bockstaele E. J. (2020). ). The Locus Coeruleus- Norepinephrine System in Stress and Arousal: Unraveling Historical, Current, and Future Perspectives. *Front. Psychiatry* 11:601519. 10.3389/fpsyt.2020.601519 33584368 PMC7873441

[B156] Schafe G. E. Nader K. Blair H. T. LeDoux J. E. (2001). Memory consolidation of Pavlovian fear conditioning: a cellular and molecular perspective. *Trends Neurosci.* 24 540–546. 10.1016/s0166-2236(00)01969-x 11506888

[B157] Schiff H. C. Johansen J. P. Hou M. Bush D. E. A. Smith E. K. Klein J. E. (2017). ). β-Adrenergic Receptors Regulate the Acquisition and Consolidation Phases of Aversive Memory Formation Through Distinct, Temporally Regulated Signaling Pathways. *Neuropsychopharmacology* 42 895–903. 10.1038/npp.2016.238 27762270 PMC5312069

[B158] Schiller D. Cain C. K. Curley N. G. Schwartz J. S. Stern S. A. Ledoux J. E. (2008). Evidence for recovery of fear following immediate extinction in rats and humans. *Learn. Mem.* 15 394–402. 10.1101/lm.909208 18509113 PMC2414250

[B159] Senn V. Wolff S. B. E. Herry C. Grenier F. Ehrlich I. Gründemann J. (2014). Long-range connectivity defines behavioral specificity of amygdala neurons. *Neuron* 81 428–437. 10.1016/j.neuron.2013.11.006 24462103

[B160] Sierra-Mercado D. Padilla-Coreano N. Quirk G. J. (2011). Dissociable roles of prelimbic and infralimbic cortices, ventral hippocampus, and basolateral amygdala in the expression and extinction of conditioned fear. *Neuropsychopharmacology* 36 529–538. 10.1038/npp.2010.184 20962768 PMC3005957

[B161] Silva B. A. Astori S. Burns A. M. Heiser H. van den Heuvel L. Santoni G. (2021). A thalamo-amygdalar circuit underlying the extinction of remote fear memories. *Nat. Neurosci.* 24 964–974. 10.1038/s41593-021-00856-y 34017129

[B162] Singewald N. Holmes A. (2019). Rodent models of impaired fear extinction. *Psychopharmacology* 236 21–32. 10.1007/s00213-018-5054-x 30377749 PMC6373188

[B163] Skelly M. J. Ariwodola O. J. Weiner J. L. (2017). Fear conditioning selectively disrupts noradrenergic facilitation of GABAergic inhibition in the basolateral amygdala. *Neuropharmacology* 113 231–240. 10.1016/j.neuropharm.2016.10.003 27720769 PMC5148638

[B164] Skelly M. J. Chappell A. M. Ariwodola O. J. Weiner J. L. (2016). Behavioral and neurophysiological evidence that lateral paracapsular GABAergic synapses in the basolateral amygdala contribute to the acquisition and extinction of fear learning. *Neurobiol. Learn. Mem.* 127 10–16. 10.1016/j.nlm.2015.11.006 26593151 PMC4718746

[B165] Snyder K. Wang W.-W. Han R. McFadden K. Valentino R. J. (2012). Corticotropin-releasing factor in the norepinephrine nucleus, locus coeruleus, facilitates behavioral flexibility. *Neuropsychopharmacology* 37 520–530. 10.1038/npp.2011.218 21993205 PMC3242313

[B166] Soeter M. Kindt M. (2015). An Abrupt Transformation of Phobic Behavior After a Post-Retrieval Amnesic Agent. *Biol. Psychiatry* 78 880–886. 10.1016/j.biopsych.2015.04.006 25980916

[B167] Southwick S. M. Bremner J. D. Rasmusson A. Morgan C. A. Arnsten A. Charney D. S. (1999). Role of norepinephrine in the pathophysiology and treatment of posttraumatic stress disorder. *Biol. Psychiatry* 46 1192–1204. 10.1016/s0006-3223(99)00219-x 10560025

[B168] Stafford J. M. Maughan D. K. Ilioi E. C. Lattal K. M. (2013). Exposure to a fearful context during periods of memory plasticity impairs extinction via hyperactivation of frontal-amygdalar circuits. *Learn. Mem.* 20 156–163. 10.1101/lm.029801.112 23422280 PMC3578276

[B169] Tonegawa S. Morrissey M. D. Kitamura T. (2018). The role of engram cells in the systems consolidation of memory. *Nat. Rev. Neurosci.* 19 485–498. 10.1038/s41583-018-0031-2 29970909

[B170] Totty M. S. Payne M. R. Maren S. (2019). Event boundaries do not cause the immediate extinction deficit after Pavlovian fear conditioning in rats. *Sci. Rep.* 9:9459. 10.1038/s41598-019-46010-4 31263140 PMC6603014

[B171] Tovote P. Fadok J. P. Lüthi A. (2015). Neuronal circuits for fear and anxiety. *Nat. Rev. Neurosci.* 16 317–331. 10.1038/nrn3945 25991441

[B172] Uematsu A. Tan B. Z. Ycu E. A. Cuevas J. S. Koivumaa J. Junyent F. (2017). Modular organization of the brainstem noradrenaline system coordinates opposing learning states. *Nat. Neurosci.* 20 1602–1611. 10.1038/nn.4642 28920933

[B173] Vaiva G. Ducrocq F. Jezequel K. Averland B. Lestavel P. Brunet A. (2003). Immediate treatment with propranolol decreases posttraumatic stress disorder two months after trauma. *Biol. Psychiatry* 54 947–949. 10.1016/s0006-3223(03)00412-8 14573324

[B174] Vale R. Evans D. A. Branco T. (2017). Rapid spatial learning controls instinctive defensive behavior in mice. *Curr. Biol.* 27 1342–1349. 10.1016/j.cub.2017.03.031 28416117 PMC5434248

[B175] Valentino R. J. Van Bockstaele E. (2008). Convergent regulation of locus coeruleus activity as an adaptive response to stress. *Eur. J. Pharmacol.* 583 194–203. 10.1016/j.ejphar.2007.11.062 18255055 PMC2349983

[B176] Van Bockstaele E. J. Colago E. E. Valentino R. J. (1998). Amygdaloid corticotropin-releasing factor targets locus coeruleus dendrites: substrate for the co-ordination of emotional and cognitive limbs of the stress response. *J. Neuroendocrinol.* 10 743–757. 10.1046/j.1365-2826.1998.00254.x 9792326

[B177] VanElzakker M. B. Dahlgren M. K. Davis F. C. Dubois S. Shin L. M. (2014). From Pavlov to PTSD: the extinction of conditioned fear in rodents, humans, and anxiety disorders. *Neurobiol. Learn. Mem.* 113 3–18. 10.1016/j.nlm.2013.11.014 24321650 PMC4156287

[B178] Vervliet B. Craske M. G. Hermans D. (2013). Fear extinction and relapse: state of the art. *Annu. Rev. Clin. Psychol.* 9 215–248. 10.1146/annurev-clinpsy-050212-185542 23537484

[B179] Wasserman E. A. Miller R. R. (1997). What’s elementary about associative learning? *Ann. Rev. Psychol.* 48 573–607. 10.1146/annurev.psych.48.1.573 9046569

[B180] Wessa M. Flor H. (2007). Failure of extinction of fear responses in posttraumatic stress disorder: evidence from second-order conditioning. *Am. J. Psychiatry* 164 1684–1692. 10.1176/appi.ajp.2007.07030525 17974933

[B181] Wilber A. A. Walker A. G. Southwood C. J. Farrell M. R. Lin G. L. Rebec G. V. (2011). Chronic stress alters neural activity in medial prefrontal cortex during retrieval of extinction. *Neuroscience* 174 115–131. 10.1016/j.neuroscience.2010.10.070 21044660 PMC3020264

[B182] Woods A. M. Bouton M. E. (2008). Immediate extinction causes a less durable loss of performance than delayed extinction following either fear or appetitive conditioning. *Learn. Mem.* 15 909–920. 10.1101/lm.1078508 19050163 PMC2632840

[B183] Yu K. Ahrens S. Zhang X. Schiff H. Ramakrishnan C. Fenno L. (2017). The central amygdala controls learning in the lateral amygdala. *Nat. Neurosci.* 20 1680–1685. 10.1038/s41593-017-0009-9 29184202 PMC5755715

[B184] Yuen E. Y. Liu W. Karatsoreos I. N. Feng J. McEwen B. S. Yan Z. (2009). Acute stress enhances glutamatergic transmission in prefrontal cortex and facilitates working memory. *Proc. Natl. Acad. Sci. USA* 106 14075–14079. 10.1073/pnas.0906791106 19666502 PMC2729022

[B185] Zhang J. Muller J. F. McDonald A. J. (2013). Noradrenergic innervation of pyramidal cells in the rat basolateral amygdala. *Neuroscience* 228 395–408. 10.1016/j.neuroscience.2012.10.035 23103792 PMC4586037

